# Association between lactate dehydrogenase levels and oncologic outcomes in metastatic prostate cancer: A meta‐analysis

**DOI:** 10.1002/cam4.3108

**Published:** 2020-05-26

**Authors:** Fan Li, Hui Xiang, Zisen Pang, Zejia Chen, Jinlong Dai, Shu Chen, Bin Xu, Tianyu Zhang

**Affiliations:** ^1^ Department of Urology Affiliated Hospital of Guilin Medical University Guilin Medical University Guilin China; ^2^ Department of Respiratory and Critical Care Medicine Affiliated Hospital of Guilin Medical University Guilin China; ^3^ Department of Urology Affiliated Hospital of Guilin Medical University Guilin China; ^4^ Department of Urology Affiliated Hospital of Guilin Medical University The Second Affiliated Hospital Of Guilin Medical University Guilin China

**Keywords:** lactate dehydrogenase, metastatic prostate cancer, oncologic outcome, prognosis

## Abstract

**Purpose:**

Previous studies have provided evidence of the high expression of lactate dehydrogenase (LDH) in multiple solid tumors; however, its prognostic relationship with metastatic prostate cancer (mPCa) remains controversial. We performed a meta‐analysis to better understand the prognostic potential of LDH in mPCa.

**Methods:**

In our investigation, we included PubMed, Embase, Web of Science, and Cochrane Library as web‐based resources, as well as studies published before January 2020 on the predictive value of LDH in mPCa. We independently screened the studies according to the inclusion and exclusion criteria, evaluated the quality of the literature, extracted the data, and used RevMan 5.3 and STATA12.0 software for analysis.

**Result:**

From the 38 published studies, the records of 9813 patients with mPCa were included in this meta‐analysis. We observed that higher levels of LDH in patients with mPCa were significantly associated with poorer overall survival (OS) (HR = 2.17, 95% CI: 1.91‐2.47, *P* < .00001) and progression‐free survival (PFS) (HR = 1.60, 95% CI: 1.20‐2.13, *P* = .001). The subgroup analyses indicated that the negative prognostic impact of higher levels of LDH on the oncologic outcomes of mPCa was significant regardless of ethnicity, publication year, sample size, analysis type, treatment type, age, and disease state.

**Conclusion:**

Our analysis suggested the association between a higher level of LDH and poorer OS and PFS in patients with mPCa. As a parameter that can be conveniently evaluated, the LDH levels should be included as a valuable biomarker in the management of mPCa.

## Graphical abstract

Our analysis suggested that lactate dehydrogenase might be a valuable biomarker, the LDH levels should be included as a valuable biomarker in the management of mPCa.

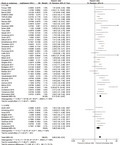

## 1 INTRODUCTION

Prostate cancer (PCa) is the most common malignancy of the male genitourinary system globally, with the highest death rate among men with neoplasias in the genitourinary system, and with nearly 1.3 million new cases and 350,000 deaths per year.^1^ Most patients have been diagnosed with metastatic prostate cancer (mPCa) during initial diagnosis,^2^ and several studies have shown that almost all patients inevitably develop castration‐resistant prostate cancer (CRPC) after treatment.^3^ To date, a variety of biomarkers have been employed in the management of PCa,^4^, ^5^, ^6^ such as the prostate‐specific antigen (PSA) or alkaline phosphatase (AKP) levels.^7^ The PSA is an internationally recognized marker of PCa. However, its influencing factors are extensive and lack specificity.^8^, ^9^, ^10^ Therefore, a search for novel biomarkers is necessary for PCa management.

Lactate dehydrogenase (LDH) is a glycolytic enzyme with five isozymes widely found in human tissues.^11^ The tumor microenvironment plays a vital role in tumor prognosis.^12^ Studies have shown that LDH plays a vital role in tumor metabolism, proliferation, invasion, and metastasis.^13^ It has been reported that the LDH levels are significantly high in several malignant tumors, and have prognostic value for various solid tumors.^14^, ^15^, ^16^ Serum LDH is easy to extract and its levels can be determined through simple processes. Multiple studies have reported an association between LDH and the oncologic outcomes in mPCa. Unfortunately, most such studies had a small sample size and the results were controversial. Therefore, we performed this meta‐analysis to comprehensively analyze the findings from such studies and to further evaluate the prognostic value of LDH in patients with mPCa.

## 2 METHODS

### 2.1 Retrieval strategy

We retrieved relevant data from PubMed, Embase, Web of Science, and Cochrane Library published during the period from their inception to January 2020. The retrieval terms used were “Metastatic PC or metastatic prostate cancer,” “LDH or lactate dehydrogenase,” and “overall survival or OS or mortality or survival or prognostic value or progression‐free survival or PFS.”

### 2.2 Inclusion and exclusion criteria

The inclusion criteria were as follows: (1) Articles published as original articles; (2) The hazard ratio (HR) and a 95% confidence interval (CI) of the levels of LDH for oncologic outcomes were provided; (3) Articles that analyzed the relationship between LDH and the oncologic outcomes in mPCa; (4) articles that were published in English.

The exclusion criteria were as follows: (1) Articles published as reports, reviews, editorials, conference abstracts; (2) Failure to provide complete information, or unclear diagnosis; (3) Animal studies; and (4) Duplicate publications, poor quality, and other unusable articles.

### 2.3 Data extraction and qualitative assessment

Two researchers independently conducted the literature screening and data extraction and consulted a third researcher for help regarding addressing inconsistencies. For data extraction, the following were included: name of first author, publication year, country, sample size, age, analysis method, oncologic outcome, treatment type, LDH cutoff level, HR, and 95% CI; the Newcastle‐Ottawa Scale (NOS) criteria was used to assess the methodological quality of the included studies.^17^ A study with a total score of 9 points and a score of 6 points was included in the study.

### 2.4 Statistical analysis

The heterogeneity of each study was evaluated using the I^2^ test. When the *P*‐value from the heterogeneity test was <.05 or the I^2^ > 50%, the random effect model was used for the pooling analysis, or a fixed‐effect model was used. In addition, a subgroup analysis was performed based on the ethnicity, publication year, sample size, analysis type, treatment type, and age to evaluate the potential sources of heterogeneity. The sensitivity analysis was also applied by eliminating a single study in a queue to identify the potential sources of heterogeneity. In addition, we evaluated the publication bias using the Begg and Egger tests.^18^, ^19^ When there was significant publication bias, we used the trim and fill method to assess whether the publication bias affected the stability of the overall estimate.^20^ A *P*‐value < .05 indicated statistical significance. For the subgroup analysis, sensitivity analysis, and determination of the publication bias, the STATA version 12.0 was used, and other statistical analyses were performed using the Review Manager version 5.3.

## 3 RESULTS

### 3.1 Search results and description

**FIGURE 1:**
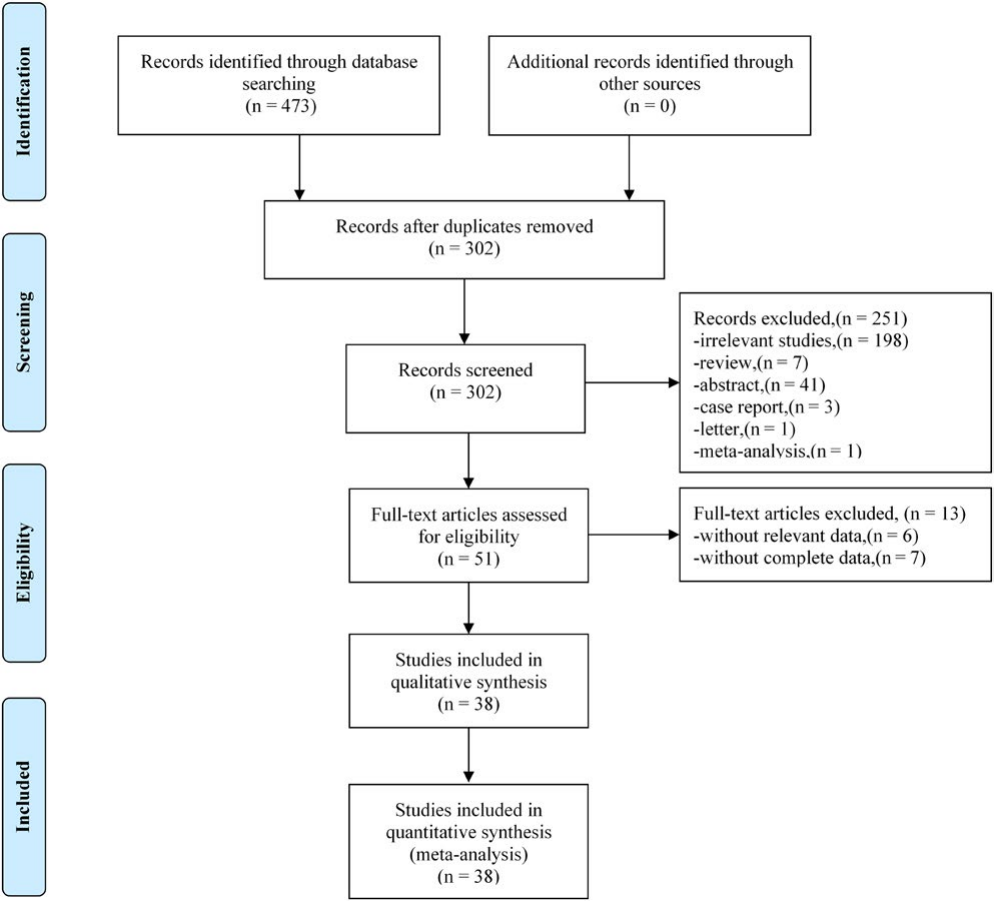
Flow diagram of studies retrieval process

A total of 473 studies were retrieved in the initial search. After the layer‐by‐layer screening, 171 duplicate and 251 irrelevant studies were excluded. Thereafter, 51 studies remained for the full‐text screening, and 13 studies were further excluded during the same. Ultimately, 38 studies were included in the meta‐analysis (Figure 1).

### 3.2 Baseline characteristics of the included studies

The characteristics of the included studies are presented in Table 1. The publication year ranged from 1998 to 2020, and there were 38 studies^21^, ^22^, ^23^, ^24^, ^25^, ^26^, ^27^, ^28^, ^29^, ^30^, ^31^, ^32^, ^33^, ^34^, ^35^, ^36^, ^37^, ^38^, ^39^, ^40^, ^41^, ^42^, ^43^, ^44^, ^45^, ^46^, ^47^, ^48^, ^49^, ^50^, ^51^, ^52^, ^53^, ^54^, ^55^, ^56^, ^57^, ^58^ with a total sample size of 9813 cases; 29 studies were conducted in European and American countries, and the rest were conducted in Asian countries; 37 studies described the relationship between LDH levels and overall survival (OS), 9 studies explored the association between LDH and progression‐free survival (PFS), 6 studies elaborated on castration‐sensitive prostate cancer (CSPC), and 33 studies discussed CRPC. All studies receive a scored from 6 to 8, suggesting that the studies were of moderate to high quality, and therefore, could be included.

**Table 1:** Baseline characteristics of included studies

First Author	Year	Country	N	Method	Outcome	Age (year)	Cut‐off (U/L)	Treatment	NOS
Furuya	1998	Japan	139	MVA	OS	75	ULN	E	8
Furuya	2003	Japan	59	MVA	OS	73	ULN	E	7
Berruti	2005	Italy	108	MVA	OS	74	398	E	6
D'AMICO	2005	USA	213	MVA/UVA	OS	72	197.3	C, E	7
TAPLIN	2005	USA	390	MVA	OS	70	208.5	E	7
Cook	2006	Canada	643	MVA/UVA	OS	71.7	454	Z	7
Saito	2007	Japan	241	MVA	OS	72.3	400	E	8
Smith	2007	USA	643	MVA/UVA	PFS	72	454	B	6
Naruse	2007	Japan	60	MVA	OS	72	ULN	E	7
Goodman	2009	USA	100	MVA/UVA	OS	71	NA	C	7
Tucci	2009	Italy	192	MVA	OS	73	NA	C, E	8
Scher	2009	USA	164	MVA	OS	70	223	C	7
Sasaki	2011	Japan	87	MVA/UVA	OS	75	250	E	8
Armstrong	2013	USA	201	MVA/UVA	OS, PFS	72	204	I	8
Schellhammer	2013	USA	512	MVA/UVA	OS	71	NA	I	7
Omlin	2013	UK	259	MVA/UVA	OS	62.1	NA	C	7
	2013	UK	183	MVA/UVA	OS	62	NA	E	7
Sonpavde	2014	USA	847	MVA/UVA	OS	68	ULN	M	7
Templeton	2014	Canada	357	MVA/UVA	OS	71	1.2*ULN	C	7
Punnoose	2015	UK	76	MVA/UVA	OS	68.9	ULN	E	7
Gravis	2015	France	385	UVA	OS	63	ULN	C, E	7
Caffo	2015	Italy	134	MVA/UVA	OS	57	382	C	7
Hung	2016	Japan	80	MVA/UVA	OS, PFS	64.6	NA	E	6
Shigeta	2016	Japan	106	MVA/UVA	OS, PFS	73	206	C	6
Kongsted	2016	Denmark	421	MVA/UVA	OS	70	ULN	C	8
Mikah	2016	Germany	84	MVA/UVA	OS	69	ULN	E	7
Sonpavde	2017	USA	794	MVA	OS	68or69	ULN	E	7
Boegemann	2017	Germany	96	MVA/UVA	OS, PFS	70	251	E	7
Buttigliero	2017	Italy	89	MVA/UVA	OS, PFS	68	ULN	C	8
Khalaf	2017	Canada	197	MVA	OS	80	ULN	E	7
Rahbar	2017	Germany	104	UVA	OS	70	225	R	6
Mehra	2018	UK	571	MVA	OS, PFS	68or69	NA	C	7
Conteduca	2018	Italy	197	MVA	OS, PFS	73	225	E	7
Okamoto	2018	Japan	339	MVA	OS, PFS	72	222	E	7
Uemura	2018	Japan	48	MVA/UVA	OS	71.2	262	C	7
Oh	2018	USA	198	MVA	OS	79	209	E	6
	2018	USA	147	MVA	OS	74	278	C	6
Vanderdoelen	2018	Netherland	45	UVA	OS	71	250	R	7
Yordanova	2020	Germany	137	MVA	OS	71	248	R	8
Shimodaira	2020	Japan	167	MVA	OS	74.8	240	E	7

Abbreviations: C, chemotherapy; E, endocrine therapy; I, immunotherapy; M, molecular targeted therapy; MVA, multivariate analysis; N, number of patients; NA, not available; NOS, Newcastle‐Ottawa Scale; OS, overall survival; PFS, progression‐free survival; R, radiotherapy; ULN, upper limit of normal; UVA, univariate analysis.

### 3.3 Results of the meta‐analyses

**FIGURE 2:**
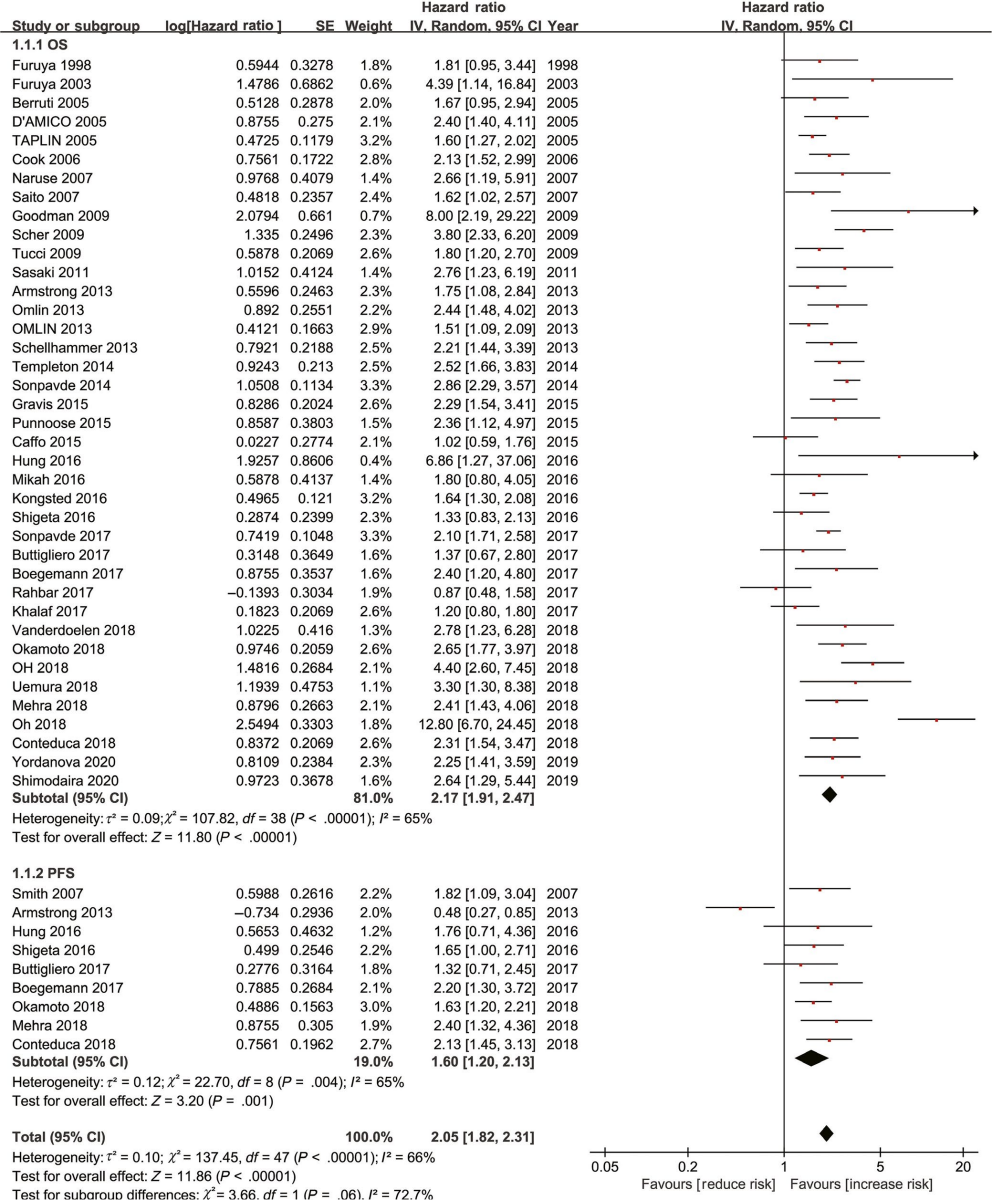
Forest plot of association between LDH and oncologic outcomes

There were 37 studies that investigated the relationship between LDH levels and OS. The heterogeneity test revealed the existence of heterogeneity in all 37 studies; hence, a random effect was used (I^2^ = 65%, *P* < .00001). The results of the meta‐analysis suggested that a higher level of LDH in patients with mPCa was significantly associated with poorer OS (HR = 2.17, 95% CI: 1.91‐2.47, *P* < .00001). In addition, nine studies evaluated the relationship between LDH levels and PFS. With observable heterogeneity in these nine studies (I^2^ = 65%, *P* = .004), a random effect was used. The results of the meta‐analysis indicated that a higher LDH level in patients with mPCa was significantly correlated with poorer PFS (HR = 1.60, 95% CI: 1.20‐2.13, *P* = .001)(Figure 2).

### 3.4 Subgroup analysis

To explore the potential sources of heterogeneity of the combined HR for the oncologic outcomes, we conducted a subgroup analysis based on ethnicity (Caucasian and Asian), publication year (before and after 2017), sample size (≥100 and <100), analysis type [multivariate analysis (MVA) and univariate analysis (UVA)], treatment type [E (endocrine therapy), C (chemotherapy) and E&C (combined endocrine and chemotherapy)], age (>>70 and ≤70 years), and disease state (CRPC and CSPC). We observed that higher LDH levels were closely associated with poorer OS in patients with mPCa. With no significant heterogeneity source observed (Table 2), a sensitivity analysis was used.

**Table 2:** Summary of overall and subgroup analyses for LDH on OS

	Studies (n)	Combined HR (95%CI)	Weight(%)	I^2^	χ^2^	*P*‐value
Overall	39	2.17 (1.91‐2.47)	100.0	65%	107.82	<.00001
Ethnicity						
Caucasian	29	2.15 (1.86‐2.50)	81.0	71%	96.37	<.00001
Asian	10	2.18 (1.72‐2.77)	19.0	21%	11.38	.25
Issuing time						
Before 2017	25	2.04 (1.78‐2.33)	64.8	50%	48.22	.002
After 2017	14	2.42 (1.83‐3.21)	35.2	77%	57.12	<.00001
Size						
≥100	29	2.12 (1.84‐2.45)	85.1	72%	100.65	<.00001
<100	10	2.42 (1.85‐3.16)	14.9	0%	5.86	.75
Method						
MVA	35	2.24 (1.96‐2.56)	60.7	64%	95.19	<.00001
UVA	22	2.44 (1.88‐3.17)	39.3	88%	174.50	<.00001
Treatment						
E	18	2.13 (1.82‐2.48)	53.7	40%	28.18	.04
C	11	2.39 (1.66‐3.44)	35.0	84%	60.63	<.00001
E&C	3	2.11 (1.64‐2.71)	11.3	0%	0.97	.61
Age (y)						
>70	22	2.46 (2.05‐2.96)	52.6	59%	51.18	.0002
≤70	17	1.89 (1.58‐2.26)	47.4	68%	49.86	<.0001
Disease state						
CRPC	33	2.16 (1.87‐2.49)	87.6	69%	103.08	<.0001
CSPC	6	2.23 (1.75‐2.85)	12.4	0%	4.37	.5

Abbreviations: C, chemotherapy; CI, confidence interval; CRPC, castration‐resistant prostate cancer; CSPC, castration‐sensitive prostate cancer; E, endocrine therapy; MVA, multivariate analysis; UVA, univariate analysis.

**FIGURE 3:**
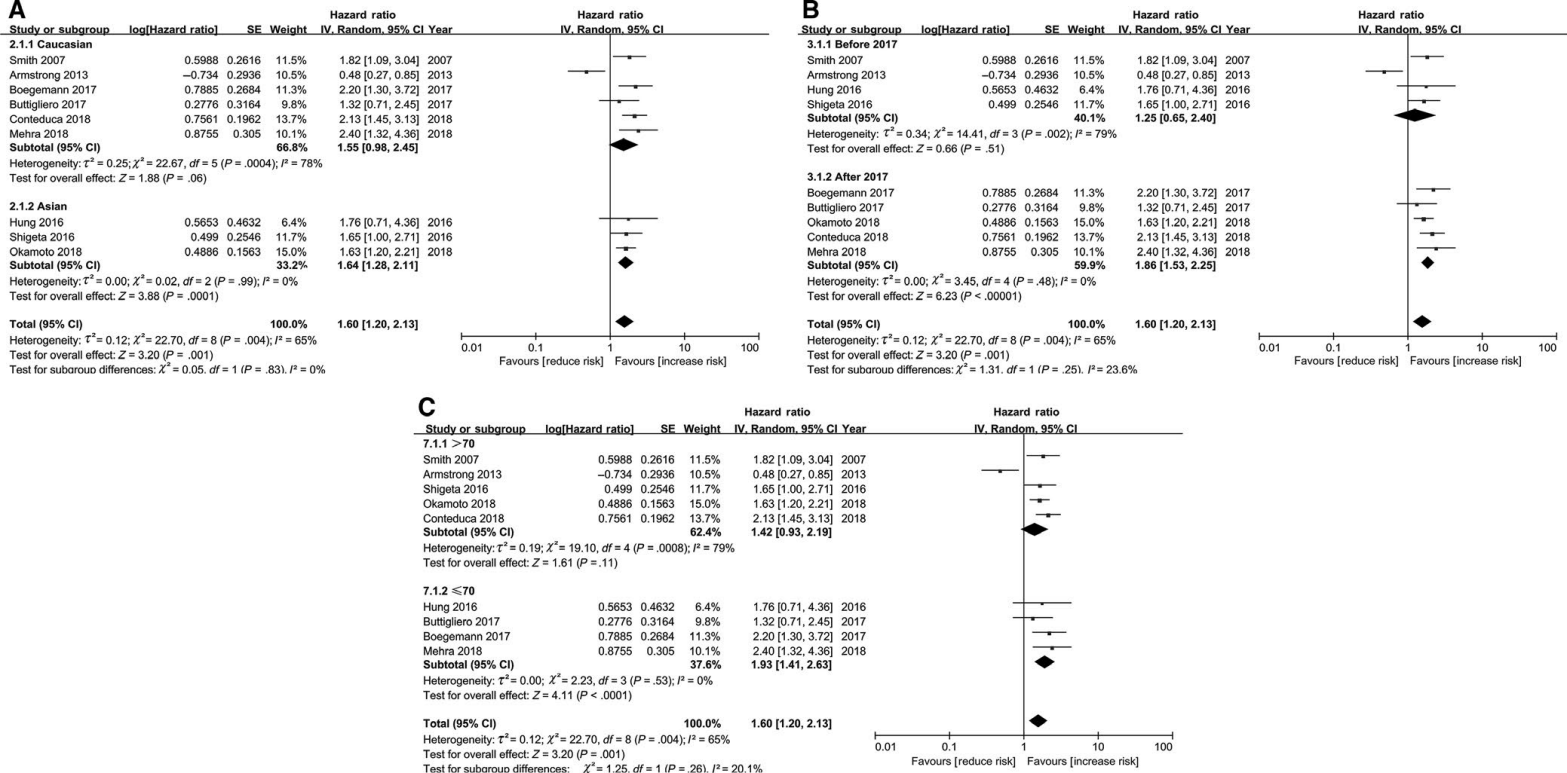
Forest plot of association between LDH and PFS. A: Association between LDH and PFS in ethnicity. B: Association between LDH and PFS in publication year. C: Association between LDH and PFS in age

In addition, the result indicated that the LDH levels were significantly associated with poorer PFS in patients with mPCa (Table 3). Furthermore, it was observed that there was one subgroup in which the heterogeneity of the combined HR for PFS was removed, suggesting that the treatment type might be the primary source of heterogeneity of the combined HR for PFS. From the subgroup analysis for PFS, the results of the subgroup for ethnicity (Caucasian), publication year (before 2017), and age (>70) indicated that higher LDH levels were not related to poorer PFS in patients with mPCa, with all *P*‐values >.05 (Figure 3).

**Table 3:** Summary of overall and subgroup analyses for LDH on PFS

	Studies (n)	Combined HR (95%CI)	Weight (%)	I^2^	χ^2^	*P*‐value
Overall	9	1.60 (1.20‐2.13)	100.0	65%	22.7	.004
Ethnicity						
Caucasian	6	1.55 (0.98‐2.45)	66.8	78%	22.67	.0004
Asian	3	2.18 (1.72‐2.77)	19.0	21%	0.02	.99
Issuing time						
Before 2017	4	1.25 (0.65‐2.40)	40.1	79%	14.41	.002
After 2017	5	1.86 (1.53‐2.25)	59.9	0%	3.45	.48
Size						
≥100	6	1.54 (1.05‐2.26)	72.5	76%	20.97	.0008
<100	3	1.77 (1.23‐2.56)	27.5	0%	1.52	.47
Method						
MVA	9	1.60 (1.20‐2.13)	64.6	65%	22.70	.004
UVA	5	1.74 (1.12‐2.71)	35.8	72%	14.21	.007
Treatment						
E	4	1.86 (1.51‐2.30)	70.3	0%	1.60	.66
C	3	1.73 (1.25‐2.40)	29.7	0%	1.92	.38
Age (y)						
>70	5	1.42 (0.93‐2.19)	62.4	79%	19.10	.0008
≤70	4	1.93 (1.41‐2.63)	37.6	0%	2.23	.53

Abbreviations: C, chemotherapy; CI, confidence interval; E, endocrine therapy; MVA, multivariate analysis; UVA, univariate analysis.

### 3.5 Sensitivity analysis

**FIGURE 4:**
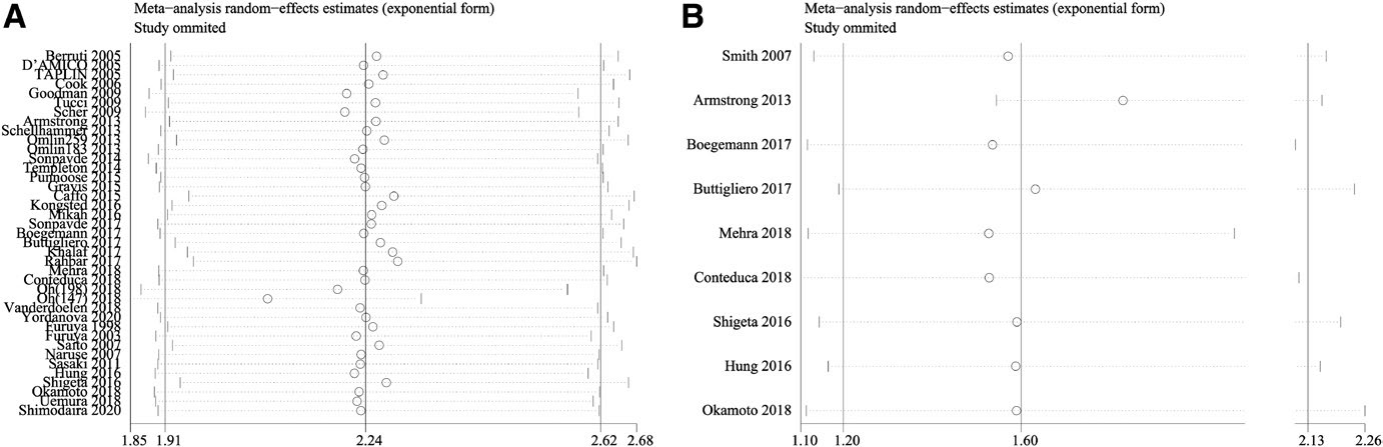
Sensitivity analysis of oncologic outcomes. A.Sensitivity analysis of OS. B,Sensitivity analysis of PFS

The sensitivity analysis was performed to determine the source of heterogeneity, as well as to confirm the stability of the combined HR for oncologic outcomes. By eliminating single studies in a queue, we observed that the heterogeneity of the combined HR for OS was removed after excluding a study^44^ (I^2^ = 48%, HR = 2.03, 95% CI: 1.86‐2.26) (Figure 4A), suggesting that this study might have been the primary source of heterogeneity of the combined HR for OS. There was no significant change in HR before and after the exclusion, indicating that the combined HR for OS was robust.

Although the treatment type might have been the primary source of heterogeneity of the combined HR for PFS, we still conducted a sensitivity analysis to determine whether the combined HR for PFS was robust. By excluding a single study in a queue, we observed that the heterogeneity of the combined HR for PFS was removed when a specific study^34^ was excluded (I^2^ = 0%, HR = 1.82, 95% CI: 1.54‐2.16) (Figure 4B), indicating that this study might have been the primary source of heterogeneity of the combined HR for OS. There was no significant change in HR before and after the exclusion, suggesting that the combined HR for PFS was robust.

### 3.6 Publication bias

**FIGURE 5:**
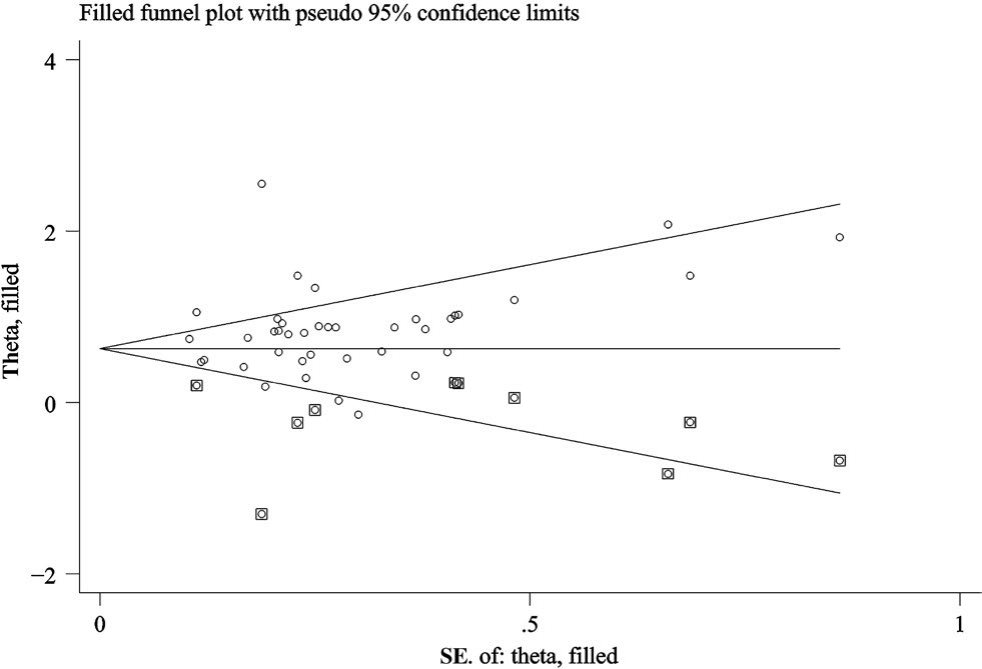
Funnel plot of trim and fill analysis

The Begg's funnel plot and Egger's tests were performed to assess the publication bias in this meta‐analysis (Table 4). The Begg's funnel plot showed symmetry, and the Egger's test suggested that there was no significant publication bias for PFS. For OS, although the Begg's funnel plot showed asymmetry and the Egger's test indicated that there was no significant publication bias, we still employed the trim and fill method to estimate the stability of the combined HR for OS. Moreover, the Begg's test might generate false positives.^59^ The results indicated that the adjusted funnel plots for OS became symmetrical (Figure 5), and that the combined HR (HR = 1.871, 95% CI: 1.561‐2.642) for OS only changed negligibly after the trim and fill method was applied, indicating the stability and reliability of our analysis.

**Table 4:** Publication bias of OS and PFS

Group	*P*‐value (Begg's test)	*P*‐value (Egger's test)
OS	.045	.478
PFS	.917	.459

Abbreviations: OS, overall survival; PFS, progression‐free survival.

## 4 DISCUSSION

In this meta‐analysis, we assessed the prognostic value of LDH in patients with mPCa by measuring the oncologic outcomes. The results showed that higher levels of LDH are associated with poorer OS and PFS in patients with mPCa (the risk of poorer OS and PFS associated with higher LDH levels is 117% and 60% greater than those with lower levels of LDH, respectively), suggesting that LDH plays a crucial prognostic role in the development of mPCa. The subgroup analysis, sensitivity analysis, publication bias determination method, and the trim and fill method adopted in our study all indicate that the combined HR for oncologic outcomes is stable and reliable.

The results of the subgroup analysis suggested the association between patient age and the levels of LDH which may affect the OS. The elderly (HR = 2.46, 95% CI: 2.05‐2.96) were at a greater risk than the younger patients, which might be attributed to the condition of the patient and the shorter life expectancy. The group PFS revealed an opposite outcome, and the result might be attributed to the adverse effects of higher LDH levels in the growth of the lower age subgroup over time, which subsequently increases the risk of disease progression. Concurrently, from the subgroup analysis of PFS, we observed that the subgroups of ethnicity (Caucasian), publication year (before 2017), and age (>70) showed that higher LDH levels were not related to poorer PFS in patients with mPCa. We believe this may have resulted from the absence of studies on the relationship between LDH levels and PFS in mPCa. Meanwhile, a large number of prospective studies are required to confirm this. In addition, there is an association between the treatment type and the levels of LDH expression that may affect the OS. The chemotherapy group (HR = 2.39, 95% CI: 2.66‐3.44) had a greater risk of poor OS than the endocrine and combination therapy groups (HR = 2.13, 95% CI: 1.82‐2.48, HR = 2.11, 95% CI: 1.64‐2.71). We inferred that these might be related to the side effects of chemotherapy that are more harmful to the human body and patient intolerance. Moreover, the results of the disease state subgroup (CRPC and CSPC) showed that higher LDH levels were significantly associated with poorer OS in patients with mPCa (HR = 2.16, 95% CI: 1.87‐2.49, HR = 2.23, 95% CI: 1.75‐2.85). In other words, LDH might be a potential biomarker for treatment selection as well as for PCa. In addition, the results of the subgroup analysis revealed that the HRs of oncologic outcomes of studies published after 2017 were higher than of those published before 2017. It is speculated the incidence of PCa has increased and its detection rate has increased as well due to advancements in medical diagnostics. The comprehensive management of PCa is not completely systematic and does not yet involve individualized clinical guidance.

Abnormally enhanced glycolytic metabolism is one of the significant biological characteristics of tumor cells. The production of lactic acid during glycolysis may promote tumor development.^60^ Lactate dehydrogenase catalyzes the reversible reaction of the conversion of pyruvate to lactic acid, which plays a critical role in glycolysis in tumor cells.^61^ Lactate dehydrogenase is a key enzyme in glycolysis and is associated with the survival and proliferation of 231 types of oncogenic cells.^62^ Although multiple studies have reported that LDH is related to the prognosis of several solid tumors, the specific mechanism underlying the process remains unclear and may be related to the Warburg effect.^63^, ^64^

This meta‐analysis offers several advantages. First, the analysis increased persuasion of the current evidence by providing a large sample size. Second, the studies selected have an encouraging representation, as studies conducted in nine countries were included. Furthermore, both the sensitivity analysis and the trim and fill method indicated that the result was robust. However, this study also has certain limitations. First, although there was no significant publication bias, most of the included studies were designed retrospectively, and therefore, more prospective studies are required to validate our analysis. Second, certain negative results might have remained unpublished, which may have led to a publication bias. Finally, the cutoff values were used to define the higher LDH levels, although the findings of the included studies were inconsistent with respect to this parameter; this would make it difficult for doctors to take clinical decisions based on LDH levels. Meanwhile, the LDH levels could have been affected by other factors, such as hepatobiliary disease, lymphoma, and heart disease among others. Some of the included studies did not distinctly state whether mPCa patients with these conditions were excluded. Therefore, a more elaborate study design and an extended follow‐up are still required to explore the prognostic value of LDH in mPCa.

## 5 CONCLUSIONS

Our meta‐analysis revealed that patients of mPCa with high LDH expression had poorer oncologic outcomes than those with low expression, with significant statistical differences. LDH is a prognostic biomarker in mPCa, and plays an important role in the proliferation of tumor cells. Moreover, the subgroup analysis confirmed that LDH is a useful prognostic factor in patients with CRPC and CSPC. Based on this, we recommend the use of LDH as a valuable biomarker in the management of mPCa.

## CONFLICT OF INTEREST

All the authors have no conflicts of interest.

## AUTHOR CONTRIBUTIONS

Fan Li and Tianyu Zhang contributed to the designation of this study. Fan Li, Hui Xiang, and Zisen Pang contributed to literature research. Zejia Chen and Jinlong Dai contributed to data extraction. Fan Li and Shu Chen contributed to the writing of the manuscript. Fan Li and Bin Xu performed the statistical analysis. All the authors contributed to and have approved the final manuscript.

## Data Availability

All data generated or analyzed during this study are included in this article.

## References

[1] Bray F , Ferlay J , Soerjomataram I , et al. Global cancer statistics 2018: GLOBOCAN estimates of incidence and mortality worldwide for 36 cancers in 185 countries. CA Cancer J Clin. 2018;68(6):394‐424. 30207593 10.3322/caac.21492

[2] Ma CG , Ye DW , Li CL , et al. Epidemiological characteristics of prostate cancer and analysis of advanced first‐line endocrine therapy. Chinese J Surg. 2008;46(12):921–925. 19035151

[3] Hellerstedt BA , Pienta KJ . The current state of hormonal therapy for prostate cancer. CA Cancer J Clin. 2002;52(3):154–179. 12018929 10.3322/canjclin.52.3.154

[4] Chi KN , Kheoh T , Ryan CJ , et al. A prognostic index model for predicting overall survival in patients with metastatic castration‐resistant prostate cancer treated with abiraterone acetate after docetaxel. Ann Oncol. 2016;27(3):454–460. 26685010 10.1093/annonc/mdv594 PMC4769990

[5] Halabi S , Lin C‐Y , Kelly WK , et al. Updated prognostic model for predicting overall survival in first‐line chemotherapy for patients with metastatic castration‐resistant prostate cancer. J Clin Oncol. 2014;32(7):671–677. 24449231 10.1200/JCO.2013.52.3696 PMC3927736

[6] Armstrong AJ , Garrett‐Mayer E , de Wit Ronald , et al. Prediction of survival following first‐line chemotherapy in men with castration‐resistant metastatic prostate cancer. Clin Cancer Res. 2010;16(1):203–211. 20008841 10.1158/1078-0432.CCR-09-2514

[7] Shariat SF , Semjonow A , Lilja H , et al. Tumor markers in prostate cancer I: Blood‐based markers. Acta Oncol. 2011;50:61‐75. 10.3109/0284186X.2010.542174 PMC3571678 21604943

[8] Catalona WJ , Partin AW , Slawin KM , et al. Use of the percentage of free prostate‐specific antigen to enhance differentiation of prostate cancer from benign prostatic disease: a prospective multicenter clinical trial. JAMA. 1998;279(19):1542‐1547. 9605898 10.1001/jama.279.19.1542

[9] Crawford ED , Rove KO , Trabulsi EJ , et al. Diagnostic performance of PCA3 to detect prostate cancer in men with increased prostate specific antigen: a prospective study of 1,962 cases. J Urol. 2012;188(5):1726–1731. 22998901 10.1016/j.juro.2012.07.023

[10] Chen R , Huang YR , Cai XB , et al. Age‐specific cutoff value for the application of percent free prostate‐specific antigen (PSA) in Chinese men with serum PSA levels of 4.0‐10.0 ng/ml. PLoS ONE. 2015;10(6):e0130308. 26091007 10.1371/journal.pone.0130308 PMC4474838

[11] Flores A , Sandoval‐Gonzalez S , Takahashi R , et al. Increased lactate dehydrogenase activity is dispensable in squamous carcinoma cells of origin. Nat Commun. 2019;10(1):91. 30626875 10.1038/s41467-018-07857-9 PMC6327029

[12] Fridman WH , Zitvogel L , Sautès–Fridman , et al. The immune contexture in cancer prognosis and treatment. Nat Rev Clin Oncol. 2017;14(12):717–734. 28741618 10.1038/nrclinonc.2017.101

[13] Koukourakis MI , Giatromanolaki A , Sivridis E , Gatter KC , Harris AL . Lactate dehydrogenase 5 expression in operable colorectal cancer: strong association with survival and activated vascular endothelial growth factor pathway—a report of the Tumour Angiogenesis Research Group. J Clin Oncol. 2006;24(26):4301‐4308. 16896001 10.1200/JCO.2006.05.9501

[14] Motzer RJ , Mazumdar M , Bacik J , et al. Survival and prognostic stratification of 670 patients with advanced renal cell carcinoma. J Clin Oncol. 1999;17(8):2530–2530. 10561319 10.1200/JCO.1999.17.8.2530

[15] Balch CM , Soong SJ , Atkins MB , et al. An evidence‐based staging system for cutaneous melanoma. CA Cancer J Clin. 2004;54:131–149. 15195788 10.3322/canjclin.54.3.131

[16] Chibaudel B , Bonnetain F , Tournigand C , et al. Simplified prognostic model in patients with oxaliplatin‐based or irinotecan‐based first‐line chemotherapy for metastatic colorectal cancer: a GERCOR study. Oncologist. 2011;16(9):1228–1238. 21859820 10.1634/theoncologist.2011-0039 PMC3228179

[17] Stang A . Critical evaluation of the Newcastle‐Ottawa scale for the assessment of the quality of nonrandomized studies in meta‐analyses. Eur J Epidemiol. 2010;25(9):603‐605. 20652370 10.1007/s10654-010-9491-z

[18] Begg CB , Mazumdar M . Operating characteristics of a rank correlation test for publication bias. Biometrics. 1994;50(4):1088‐1101. 7786990

[19] Egger M , Smith GD , Schneider M , Minder C . Bias in meta‐analysis detected by a simple, graphical test. BMJ. 1997;315(7109):629‐634. 9310563 10.1136/bmj.315.7109.629 PMC2127453

[20] Duval S , Tweedie R . Trim and fill: a simple funnel‐plot‐based method of testing and adjusting for publication bias in meta‐analysis. Biometrics. 2000;56(2):455‐463. 10877304 10.1111/j.0006-341x.2000.00455.x

[21] Furuya Y , Akimoto S , Akakura K , et al. Response of prostate‐specific antigen after androgen withdrawal and prognosis in men with metastatic prostate cancer. Urol Int. 1998;60(1):28‐32. 9519418 10.1159/000030199

[22] Furuya Y , Nagakawa O , Fuse H . Prognostic significance of changes in short‐term prostate volume and serum prostate‐specific antigen after androgen withdrawal in men with metastatic prostate cancer. Urol Int. 2003;70(3):195‐199. 12660456 10.1159/000068769

[23] Berruti A , Mosca A , Tucci M , et al. Independent prognostic role of circulating chromogranin A in prostate cancer patients with hormone‐refractory disease. Endocr Relat Cancer. 2005;12(1):109‐117. 15788643 10.1677/erc.1.00876

[24] D'Amico AV , Chen MH , Cox MC , et al. Prostate‐specific antigen response duration and risk of death for patients with hormone‐refractory metastatic prostate cancer. Urology. 2005;66(3):571‐576. 16140080 10.1016/j.urology.2005.03.083

[25] Taplin ME , George DJ , Halabi S , et al. Prognostic significance of plasma chromogranin a levels in patients with hormone‐refractory prostate cancer treated in Cancer and Leukemia Group B 9480 study. Urology. 2005;66(2):386‐391. 16098367 10.1016/j.urology.2005.03.040

[26] Cook RJ , Coleman R , Brown J , et al. Markers of bone metabolism and survival in men with hormone‐refractory metastatic prostate cancer. Clin Cancer Res. 2006;12:3361‐3367. 16740758 10.1158/1078-0432.CCR-06-0269

[27] Saito T , Hara N , Kitamura Y , Komatsubara S . Prostate‐specific antigen/prostatic acid phosphatase ratio is significant prognostic factor in patients with stage IV prostate cancer. Urology. 2007;70(4):702‐705. 17991541 10.1016/j.urology.2007.05.019

[28] Smith MR , Cook RJ , Coleman R , et al. Predictors of skeletal complications in men with hormone‐refractory metastatic prostate cancer. Urology. 2007;70(2):315‐319. 17826496 10.1016/j.urology.2007.03.071 PMC3047396

[29] Naruse K , Yamada Y , Aoki S , et al. Lactate dehydrogenase is a prognostic indicator for prostate cancer patients with bone metastasis. Hinyokika kiyo Acta urologica Japonica. 2007;53(5):287‐292. 17561711

[30] Goodman OB , Fink LM , Symanowski JT , et al. Circulating tumor cells in patients with castration‐resistant prostate cancer baseline values and correlation with prognostic factors. Cancer Epidemiol Biomark Prev. 2009;18(6):1904‐1913. 10.1158/1055-9965.EPI-08-1173 19505924

[31] Tucci M , Mosca A , Lamanna G , et al. Prognostic significance of disordered calcium metabolism in hormone‐refractory prostate cancer patients with metastatic bone disease. Prostate cancer and prostatic diseases. 2009;12(1):94‐99. 18332901 10.1038/pcan.2008.10

[32] Scher HI , Jia X , de Bono JS , et al. Circulating tumour cells as prognostic markers in progressive, castration‐resistant prostate cancer: a reanalysis of IMMC38 trial data. Lancet Oncol. 2009;10(3):233‐239. 19213602 10.1016/S1470-2045(08)70340-1 PMC2774131

[33] Sasaki T , Onishi T , Hoshina A . Nadir PSA level and time to PSA nadir following primary androgen deprivation therapy are the early survival predictors for prostate cancer patients with bone metastasis. Prostate cancer and prostatic diseases. 2011;14(3):248‐252. 21502970 10.1038/pcan.2011.14

[34] Armstrong A , Häggman M , Stadler W , et al. Long‐term survival and biomarker correlates of tasquinimod efficacy in a multicenter randomized study of men with minimally symptomatic metastatic castration‐resistant prostate cancer. Clin Cancer Res. 2013;19(24):6891‐6901. 24255071 10.1158/1078-0432.CCR-13-1581 PMC4251453

[35] Schellhammer PF , Chodak G , Whitmore JB , et al. Lower baseline prostate‐specific antigen is associated with a greater overall survival benefit from sipuleucel‐T in the Immunotherapy for Prostate Adenocarcinoma Treatment (IMPACT) trial. Urology. 2013;81(6):1297‐1302. 23582482 10.1016/j.urology.2013.01.061

[36] Omlin A , Pezaro C , Mukherji D , et al. Improved survival in a cohort of trial participants with metastatic castration‐resistant prostate cancer demonstrates the need for updated prognostic nomograms. Eur Urol. 2013;64(2):300‐306. 23313031 10.1016/j.eururo.2012.12.029

[37] Sonpavde G , Pond GR , Armstrong AJ , et al. Prognostic impact of the neutrophil‐to‐lymphocyte ratio in men with metastatic castration‐resistant prostate cancer. Clinical genitourinary cancer. 2014;12(5):317‐324. 24806399 10.1016/j.clgc.2014.03.005

[38] Templeton AJ , Pezaro C , Omlin A , et al. Simple prognostic score for metastatic castration‐resistant prostate cancer with incorporation of neutrophil‐to‐lymphocyte ratio. Cancer. 2014;120(21):3346‐3352. 24995769 10.1002/cncr.28890

[39] Punnoose EA , Ferraldeschi R , Szafer‐Glusman E , et al. PTEN loss in circulating tumour cells correlates with PTEN loss in fresh tumour tissue from castration‐resistant prostate cancer patients. Br J Cancer. 2015;113(8):1225‐1233. 26379078 10.1038/bjc.2015.332 PMC4647881

[40] Gravis G , Boher JM , Fizazi K , et al. Prognostic factors for survival in noncastrate metastatic prostate cancer: validation of the glass model and development of a novel simplified prognostic model. Eur Urol. 2015;68(2):196‐204. 25277272 10.1016/j.eururo.2014.09.022

[41] Caffo O , Ortega C , Di Lorenzo G , et al. Clinical outcomes in a contemporary series of “young” patients with castration‐resistant prostate cancer who were 60 years and younger. Urol Oncol. 2015;33(6):265.e15‐265.e21. 10.1016/j.urolonc.2015.02.016 25907622

[42] Hung J , Taylor AR , Divine GW , et al. The effect of time to castration resistance on outcomes with abiraterone and enzalutamide in metastatic prostate cancer. Clin Cancer Res. 2016;14(5):381‐388. 10.1016/j.clgc.2016.03.021 27157640

[43] Shigeta K , Kosaka T , Kitano S , et al. High absolute monocyte count predicts poor clinical outcome in patients with castration‐resistant prostate cancer treated with docetaxel chemotherapy. Ann Surg Oncol. 2016;23(12):4115‐4122. 27364499 10.1245/s10434-016-5354-5

[44] Kongsted P , Svane IM , Lindberg H , Sengeløv L . Predictors of chemotherapy‐induced toxicity and treatment outcomes in elderly versus younger patients with metastatic castration‐resistant prostate cancer. Clin Cancer Res. 2016;14(6):e559‐e568. 10.1016/j.clgc.2016.03.018 27102406

[45] Mikah P , Krabbe L‐M , Eminaga O , et al. Dynamic changes of alkaline phosphatase are strongly associated with PSA‐decline and predict best clinical benefit earlier than PSA‐changes under therapy with abiraterone acetate in bone metastatic castration resistant prostate cancer. BMC Cancer. 2016;16(1):214. 26975660 10.1186/s12885-016-2260-y PMC4790058

[46] Sonpavde G , Pond G , Templeton A , Kwon ED , De Bono J . Impact of single‐agent daily prednisone on outcomes in men with metastatic castration‐resistant prostate cancer. Prostate Cancer Prostatic Dis. 2017;20(1):67‐71. 27670718 10.1038/pcan.2016.44

[47] Martin B , Katrin S , Stefan T , et al. The role of the neutrophil to lymphocyte ratio for survival outcomes in patients with metastatic castration‐resistant prostate cancer treated with abiraterone. Int J Mol Sci. 2017;18(2):380. 10.3390/ijms18020380 PMC5343915 28208664

[48] Buttigliero C , Pisano C , Tucci M , et al. Prognostic impact of pretreatment neutrophil‐to‐lymphocyte ratio in castration‐resistant prostate cancer patients treated with first‐line docetaxel. Acta Oncol. 2017;56(4):555‐562. 28068151 10.1080/0284186X.2016.1260772

[49] Khalaf DJ , Avilés CM , Azad AA , et al. A prognostic model for stratifying clinical outcomes in chemotherapy‐naive metastatic castration‐resistant prostate cancer patients treated with abiraterone acetate. Can Urol Assoc J. 2018;12(2):E47. 29381456 10.5489/cuaj.4600 PMC5937410

[50] Rahbar K , Boegemann M , Yordanova A , et al. PSMA targeted radioligand therapy in metastatic castration resistant prostate cancer after chemotherapy, abiraterone and/or enzalutamide. A retrospective analysis of overall survival. Eur J Nucl Med Mol Imaging. 2018;45(1):12‐19. 29026946 10.1007/s00259-017-3848-4

[51] Mehra N , Dolling D , Sumanasuriya S , et al. Plasma cell‐free DNA concentration and outcomes from taxane therapy in metastatic castration‐resistant prostate cancer from two phase III trials (FIRSTANA and PROSELICA). Eur Urol. 2018;74(3):283‐291. 29500065 10.1016/j.eururo.2018.02.013 PMC6090941

[52] Conteduca V , Scarpi E , Salvi S , et al. Plasma androgen receptor and serum chromogranin A in advanced prostate cancer. Sci Rep. 2018;8(1):1‐8. 30337589 10.1038/s41598-018-33774-4 PMC6194135

[53] Okamoto T , Hatakeyama S , Narita S , et al. Impact of nutritional status on the prognosis of patients with metastatic hormone‐naive prostate cancer: a multicenter retrospective cohort study in Japan. World J Urol. 2019;37(9):1827‐1835. 30511214 10.1007/s00345-018-2590-2

[54] Uemura K , Miyoshi Y , Kawahara T , et al. Prognostic value of an automated bone scan index for men with metastatic castration‐resistant prostate cancer treated with cabazitaxel. BMC Cancer. 2018;18(1):501. 29716525 10.1186/s12885-018-4401-y PMC5930579

[55] Oh WK , Cheng WY , Miao R , et al. Real‐world outcomes in patients with metastatic castration‐resistant prostate cancer receiving second‐line chemotherapy versus an alternative androgen receptor‐targeted agent (ARTA) following early progression on a first‐line ARTA in a US community oncology setting. Urol Oncol. 2018;36(11):500.e1‐500.e9. 10.1016/j.urolonc.2018.08.002 30201382

[56] van der Doelen MJ , Kuppen MC , Jonker MA , et al. 223Ra therapy in patients with advanced castration‐resistant prostate cancer with bone metastases: lessons from daily practice. Clin Nucl Med. 2018;43(1):9‐16. 29166331 10.1097/RLU.0000000000001904

[57] Yordanova A , Linden P , Hauser S , et al. The value of tumor markers in men with metastatic prostate cancer undergoing [177Lu] Lu‐PSMA therapy. Prostate. 2020;80(1):17‐27. 31579967 10.1002/pros.23912

[58] Shimodaira K , Nakashima J , Nakagami Y , et al. Prognostic value of platelet counts in patients with metastatic prostate cancer treated with endocrine therapy. Urol J. 2020;17(1):42‐49. 30882158 10.22037/uj.v0i0.4735

[59] Seagroatt V , Stratton I . Bias in meta‐analysis detected by a simple, graphical test. Test had 10% false positive rate. BMJ. 1998;316(7129):470. PMC2665628 9492688

[60] Feng Y , Xiong Y , Qiao T , Li X , Jia L , Han Y . Lactate dehydrogenase A: A key player in carcinogenesis and potential target in cancer therapy. Cancer Med. 2018;7(12):6124‐6136. 30403008 10.1002/cam4.1820 PMC6308051

[61] Rani R , Kumar V . Recent update on human lactate dehydrogenase enzyme 5 (h LDH5) inhibitors: a promising approach for cancer chemotherapy: miniperspective. J Med Chem. 2016;59(2):487‐496. 26340601 10.1021/acs.jmedchem.5b00168

[62] Xie H , Hanai J‐I , Ren J‐G , et al. Targeting lactate dehydrogenase‐a inhibits tumorigenesis and tumor progression in mouse models of lung cancer and impacts tumor‐initiating cells. Cell Metab. 2014;19(5):795‐809. 24726384 10.1016/j.cmet.2014.03.003 PMC4096909

[63] Bachelot T , Ray‐Coquard I , Catimel G , et al. Multivariable analysis of prognostic factors for toxicity and survival for patients enrolled in phase I clinical trials. Ann Oncol. 2000;11(2):151‐156. 10761748 10.1023/a:1008368319526

[64] Cheng A , Zhang P , Wang B , et al. Aurora‐A mediated phosphorylation of LDHB promotes glycolysis and tumor progression by relieving the substrate‐inhibition effect. Nat Commun. 2019;10(1):1‐16. 31804482 10.1038/s41467-019-13485-8 PMC6895051

